# Evaluation of Safety and Efficacy of ReHub in Patients Who Underwent Primary Total Knee Arthroplasty: Study Protocol for a Randomized Controlled Trial

**DOI:** 10.29337/ijsp.138

**Published:** 2021-04-19

**Authors:** Montse Nuevo, Hadis Mahdavi, Daniel Rodríguez, Teresa Faura, Núria Fabrellas, Simone Balocco, Marco Conti, Alessandro Castagna, Salvi Prat

**Affiliations:** 1 Clinic Institute of Medical and Surgical Specialties (ICEMEQ), Knee Unit, Hospital Clínic of Barcelona, C.Villarroel 170, 08036, Barcelona, Spain; 2 Nursing and Health Sciences (PhD programme), University of Barcelona, Faculty of Medicine and Health Sciences, Bellvitge Health Sciences Campus, C.Feixa Llarga s/n, L’Hospitalet de Llobregat, 08907, Spain; 3 Bio-Sensing Solutions S.L. (DyCare), Barcelona, Spain; 4 Medicine School, Campus Casanova, University of Barcelona, C.Casanova, 143, 08036, Barcelona, Spain; 5 School of Nursing Campus Clínic, Faculty of Medicine and Health Sciences, University of Barcelona, Barcelona; University of Barcelona, C.Casanova, 143, 08036, Barcelona, Spain; 6 Department of Mathematics and Informatics, University of Barcelona, Barcelona, Spain; 7 MediSport, Human Performance Lab – Como and Varese, Italy; 8 Humanitas Clinical and Research Center, IRCSS, Rozzano (Mi), Italy; 9 Humanitas University, Department of Biomedical Sciences, Pieve Emanuele (Mi), Italy

**Keywords:** home rehabilitation, telerehabilitation, physical therapy, total knee arthroplasty, digital health

## Abstract

**Background:**

Total Knee Arthroplasty (TKA) is an intervention that can significantly improve the quality of life of patients with advanced knee osteoarthritis. Early start of rehabilitation and its continuation at home once the patient is discharged are key factors for the success of the process.

This study aims to assess the effectiveness of a home-based telerehabilitation solution (ReHub) on improving functional capacity and clinical outcomes for patients who underwent TKA.

**Methods/design:**

The study is a randomized, open-label with blinded outcome assessor, parallel assignment clinical trial with a sample size of 52 patients that is conducted according to the SPIRIT recommendations. After the TKA intervention, the patients are randomly allocated to the control group or the experimental group with a 1:1 ratio. Both groups follow a Fast Track recovery protocol which includes discharge after 2–3 days from surgery, a daily plan of 5 exercises for autonomous rehabilitation and domiciliary visits by a physiotherapist starting approximately 2 weeks after surgery. The experimental group uses the sensor-based telerehabilitation system ReHub to perform the exercises. The primary outcome measure is the range of motion of the knee. Secondary outcomes include physical performance, quality of life, pain intensity, muscle strength, treatment adherence and satisfaction with the ReHub system. The outcomes assessment is performed at hospital discharge (baseline), at stitch removal (2 weeks after baseline) and 2 weeks after stitch removal (4 weeks after baseline).

The study conforms to the guidelines of the Declaration of Helsinki and was approved by the hospital’s ethics committee.

**Discussion:**

The study will address an important gap in the evidence base by reporting the effectiveness of an affordable and low-cost home-based telerehabilitation solution in patients who underwent TKA.

**Ethics and dissemination:**

The study was approved by the hospital’s ethics committee (“Comité Ético de Investigación Clínica del HCB”, reg. HCB/2019/0571). The trial was registred at ClinicalTrials.gov (NCT04155957). The results of this study will be published in peer-reviewed journals as well as national and international conferences.

**Trial registration:**

NCT04155957 (ClinicalTrials.gov).

**Highlights:**

- Assessing a home-based telerehabilitation solution effectiveness in knee surgery.
- In situations such as the CoVid-19 pandemic, it is a resolutive intervention method.
- Telerehabilitation is an alternative to conventional face-to-face physical therapy.
- This system is far less demanding in terms of human resources.
- Range of motion assessment is the primary outcome measure.

## Background

Knee osteoarthritis (KO) is the most common joint condition and causes significant amounts of disability worldwide [1]. Total Knee Arthroplasty (TKA) is an intervention which has improved the quality of life of the patients with advanced knee osteoarthritis. In recent years, “Rapid Recovery” or “Fast-Track” strategies, have been developed and applied in TKA and other selective operations. These strategies aim to enhance post-operative recovery and reduce morbidity, functional convalescence and hospital stay lengths and costs [2,3]. Pre-operative rehabilitation and patient empowerment, as well as the early start of post-operative rehabilitation once the patient is discharged, are key factors for the success of the recovery process after a TKA [4,5].

Patients with TKA operated at some hospitals in Spain systematically undergo a specific training program a few weeks before surgery. They familiarize themselves with the surgical process and the physical exercises they shall perform before and immediately after the surgery. This allows for a better recovery and decreases the stress of surgery [6]. After hospital discharge (usually between 48h to 72h after surgery), patients continue the exercise program at home as they have been taught. However, the adherence of the patients to this program is unknown. To monitor patients’ activities, health professionals can only rely on the information provided by the patients themselves and/or their families. Approximately 2 weeks after the surgery, patients start receiving visits from a physiotherapist at home. Domiciliary rehabilitation is estimated to take 10 sessions distributed in 2 or 3 days per week, depending on the availability.

On the other hand, telerehabilitation has been rapidly expanding as an alternative or a complement to conventional face-to-face physical therapy since its development [7]. Telerehabilitation systems monitor the patients with sensors and software, allowing the therapist to intervene in the rehabilitation progress remotely. The patient feels monitored, motivated and supported [8]. Besides, comparable results are obtained to conventional outpatient physical therapy [9,10], or even face-to-face physical therapy with home rehabilitation [11], and are widely accepted by patients [9,10].

Recently Correia et al. [12] demonstrated that home telerehabilitation after TKA is feasible, engaging, and capable of maximizing clinical outcomes in comparison to conventional rehabilitation in the short and medium-term and is far less demanding in terms of human resources.

According to other recent studies, telerehabilitation shows a positive impact on patient compliance and adherence [9,11,13], which is a really important factor for a better functional recovery after TKA surgery. We must not forget the role of these innovative technologies in empowering the patient and promoting their active participation in their recovery process, a vital aspect in achieving maximum success in early and late functional outcomes [14]. It is also important to consider the patient’s opinion on the matter. There is strong evidence to support the use of virtual systems to increase patient satisfaction in patients who have undergone TKA [15]. This trial contributes to the scientific knowledge base regarding the effects of telerehabilitation.

We hypothesized that a home-based telerehabilitation program performed through the remote supervision of the patient’s performance and adherence can improve clinical outcomes compared to conventional (unsupervised) home-based rehabilitation.

Therefore, the overall objective of this study is to evaluate the effectiveness of a new telerehabilitation solution, ReHub, for the improvement of physical function and clinical outcomes following TKA. ReHub is an interactive telerehabilitation system developed by DyCare which delivers personalized home rehabilitation for Muscle-Skeletal Disorder (MSD) sufferers.

## Methods

### Study design and randomization

The study is a prospective, randomized, controlled, parallel-group, open-label with blinded assessor trial that is conducted according to the SPIRIT recommendations [16]. Following informed consent, patients are randomized (with a 1:1 allocation ratio) to a control group or an experimental group. Computer-generated randomization lists are used (using the website www.randomization.com) to sequentially distribute the patients into one of the two groups. The primary outcome measure is knee Range of Motion (ROM).

The study conforms to the guidelines of the Declaration of Helsinki, was approved by the hospital’s ethics committee (“Comité Ético de Investigación Clínica del HCB”, reg. HCB/2019/0571), and registered at the ClinicalTrials.gov website (identifier NCT04155957).

**Figure 1:**
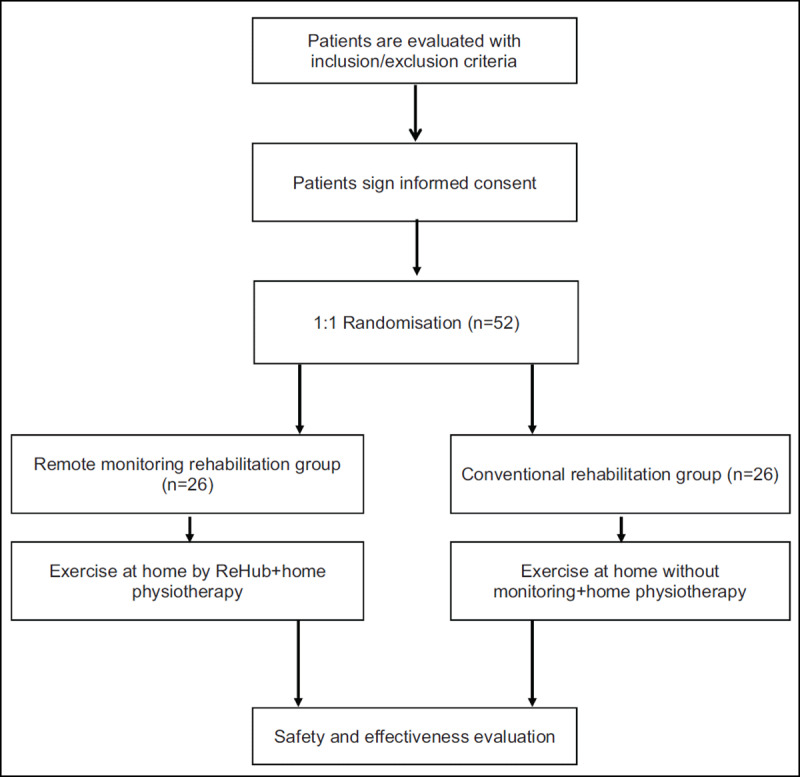
REHAPT flow diagram.

A flow diagram of the study design is shown in ***Figure 1***.

### Participants/randomization/allocation/blinding

The general outline of this open-label, parallel-assignment, controlled clinical trial with blinded evaluators is the following: candidates to a TKA with no interfering comorbidities and between 18 and 75 years old will need to sign an Informed Consent Form to be enrolled in the study. The patients then will be randomized with an allocation ratio of 1:1 into one of the two groups: the Control group, receiving the traditional home rehabilitation that patients who undergo Total Knee Arthroplasty (TKA) in a third level hospital (Rapid Recovery Program), or the Experimental group, following the same rehabilitation plan for TKA with ReHub instead of usual autonomous rehabilitation exercices.

Following the allocation of the groups, trained blinded nurses will complete all functional assessments and collect all clinical data on the Case Report Form for each patient. These evaluations will be made at the time of discharge from hospital, after two weeks and after four weeks, both during the follow-up visits.

A total of 52 patients will be recruited following a personal interview carried out by the investigators considering the following inclusion and exclusion criteria mentioned in ***Table 1***.

**Table 1:** Inclusion and Exclusion criteria.


INCLUSION CRITERIA:

1. Age of the candidates shall be between 18 and 75 years 2. Ability to understand and accept the study procedures and to sign an informed consent form 3. Good predisposition to the use of technology or availability of a caregiver providing technological support to the patient 4. Availability to move to the hospital for visits 5. The patient resides in the hospital’s area of influence

**EXCLUSION CRITERIA:**

Any type of disability that could alter the homogeneity of the study leads to exclusion as well as sensory and/or cognitive impairment or concomitant medical conditions which may affect the rehabilitation process. In addition, major medical complications occurring after surgery (e.g. surgical wound infection, suspicious of deep vein thrombosis) also lead to exclusion.


The expected recruitment rate is 2 patients per week.

### Interventions

Patients are normally discharged 48 hours after the operation with an updated physiotherapy schedule and a leaflet containing the exercises and patterns to follow. The physiotherapist makes sure that the patients (from both groups) have understood them and will perform them correctly.

The rehabilitation program duration per patient is 4 weeks (30 days) after hospital discharge. Three visits take place, one at discharge (baseline), another after 2 weeks and the final visit after 4 weeks (***Table 2***).

**Table 2:** Timepoint chart.


	RECRUITMENT	BASELINE VISIT	INTERVENTION (2 WEEKS)	FOLLOW-UP VISIT	INTERVENTION (2 WEEKS)	FINAL VISIT

**TIMEPOINT**	**-t_o_**	**t_0_**		**t_1_**		**t_2_**

Screening & Eligibility	X					

Informed consent procedure	X					

Treatment randomization	X					

ReHub training (Experimental Arm only)			X			

Timed Up-and-Go		X		X		X

Knee ROM		X		X		X

Muscle strength		X		X		X

Pain level (VAS)		X		X		X

WOMAC		X		X		X

EuroQol-5D-5L		X		X		X

Patient diary data collection (Control Arm only)						X

System Usability Scale (Experimental Arm only)						X

Adherence						X

Adverse events				X		X


The following outcome measures are collected for the patients at the visits:

- Active Knee ROM (°) with a goniometer, both flexion and extension
- Passive Knee ROM (°) with a goniometer, both flexion and extension
- Timed Get-Up-and-Go test (s)
- Quadriceps muscle strength with a dynamometer (kg)
- Hamstring muscle strength with a dynamometer (kg)
- Pain Level with a Visual Analog Scale (VAS)
- Western Ontario and McMaster Universities Osteoarthritis Index (WOMAC)
- EuroQol-5D-5L score
- Adherence to exercise program (only collected in last visit)
- System Usability Scale (only collected in last visit for experimental patients)

Both groups shall perform at home the prescribed exercises. The control group will follow the common home rehabilitation recommendations and exercise autonomously while the experimental group patients will use ReHub to perform their exercises; for both groups the exercise pattern will be the same.

In addition, and as part of the usual hospital protocol, during the last two weeks, the participants of both groups will receive a visit from a physiotherapist from an external company, 2–3 days per week, up to 10 sessions. The details of the intervention for each group are as follows:

- Experimental Group:
  A site team physiotherapist will make three home visits during the 4 weeks. In the first visit (the day after discharge), the patient receives a ReHub bag including a tablet with ReHub installed and the Exercise Kit. The site team physiotherapist explains how ReHub works and how to perform exercises. The second visit (second day after discharge) is a follow-up visit to ensure the correct use of ReHub so the patient can perform his/her exercises properly. An optional third visit can be planned for the following day at this stage if the patient has difficulties using the platform. The patient follows the exercise program with ReHub, and a site team physiotherapist monitors the patient’s progress daily through the platform.
  12–17 days after surgery, the patient attends the hospital for stitch removal and the second visit takes place. If required, in this visit the therapy is reprogrammed. The day after the second visit, a site team physiotherapist makes the last home visit to ensure no problems have arisen during the last 2 weeks.
  Around 30 days after the surgery, patients attend the hospital for the final control. In addition to the aforementioned outcome measures, patient satisfaction with ReHub is measured by the administration of the System Usability Scale as well.
- Control Group:
  The 3 main visits are the same as the experimental group and the same outcomes are measured. The control group does not receive home visits from the site team physiotherapists. Patients are asked to fill out a diary to indicate which exercises they performed and the corresponding dates. At the final visit at the hospital, in addition to collecting the aforementioned outcome measures, the patients’ exercise diary will be collected.

### Outcome Measures

#### Primary outcomes

##### 1. ROM Active and Passive

All ROM tests are conducted using a manual, plastic, 2-group goniometer with 1-degree increments. The goniometer is centred on the knee joint. The distal reference marker is the peroneal malleolus and the proximal reference point is the greater trochanter in the hip [17]. To make sure that the goniometer is centred on the knee axis point, the patient is asked to bend and extend the knee a couple of times. This axis point should not change in the flexion and extension movement. For the measurement of the active flexion ROM, the patient is sitting in a chair and is asked to bend the knee as much as possible. For passive flexion ROM, the assistant helps the patient to bend until the patient indicates that it is the maximum point.

For the active extension ROM, the patient is lying in the supine position on a bed and is asked to stretch the knee as far as possible. For passive extension ROM, the assistant helps the patient to stretch until the patient says it is the maximum point.

#### Secondary outcomes

##### 1. Timed Up-and-Go

The Timed Up-and-Go (TUG) test measures the time required for standing up from a chair, walking straight for 3 meters, turning, walking back to the chair, and sitting down [18]. The nurse is in charge of indicating when to start the test.

##### 2. Muscle strength

The maximal isometric voluntary contraction of the operated leg is assessed using a handheld dynamometer (Lafayette Manual Muscle Tester) [19]. The muscle strength test of the knee extension is performed for the quadriceps and then the knee flexion test is performed for the hamstrings. Knee extension is assessed with the participants in sitting position with their feet on the floor and with the hip at 90° of flexion.

##### 3. VAS

To assess the pain level, the patient is asked to rate the pain he/she feels on a Visual Analog Scale that ranges from 0 to 10, with 0 being «no pain» and 10 being the “worst pain imaginable” [20].

##### 4. WOMAC – EuroQoL-5D-5L

WOMAC (Western Ontario and McMaster Universities Osteoarthritis Index) [21,22] and EuroQoL-5D-5L [23] are standard questionnaires which are self-administered by the patients and will be compared with the baseline later in the analysis to explore the improvements in the quality of life of the patients in both groups of the study.

##### 5. Adherence

The adherence to the home exercise program is measured by obtaining the percentage of exercises done from the ReHub database for the experimental group and from the exercise diary filled by control patients in the control group.

##### 6. Satisfaction and Safety of the ReHub System

The experimental patients’ satisfaction with ReHub is measured with the results of the System Usability Scale (SUS) [24]. The questionnaire is also self-administered.

The Physiotherapist-Patient interaction for the experimental group is also measured through the number of messages exchanged between them within ReHub’s chat feature.

The safety of the system will be evaluated with the rate of adverse events reported during the study. Examples of adverse events are minor cutaneous injuries, indentations on soft body parts and software failures.

### ReHub telerehabilitation system

ReHub is a digital platform for physical rehabilitation that offers personalized design and monitoring of therapeutic exercise programs to recover the functional capacity of the musculoskeletal system.

The solution is composed of two main pillars; a cloud platform and an exercise kit that includes an inertial motion sensor. The cloud platform establishes communication between the patient and the healthcare professionals in charge of their rehabilitation. It allows physical therapists to create a rehabilitation program specifically tailored to each patient’s condition. A dashboard allows the professional to follow the progress of the patient, see the results of the exercises performed and adapt the program remotely.

Patients use the cloud platform to perform the exercises in their rehabilitation program with the help of DyCare’s proprietary wearable sensor that captures 3D motion data. The sensor is integrated into different exercise tools that help the patient performing different kinds of exercises make up the rest of the kit, though only a body strap is used in this trial as the exercises in the rehabilitation program designed by the hospital do not require additional tools. The sensor records biomechanical parameters such as range of motion and speed in real-time when used on the indicated body part while exercising. When patients do their prescribed exercises at home, intelligent algorithms deliver real-time biofeedback through a User Interface and a Virtual Coach. The results can be viewed by the physiotherapist to follow the progress of the patient, adapt the program remotely if needed or chat with them with the online messaging module through the platform.

### Sample size estimation and statistical analysis

Sample size estimation was performed considering active knee flexion Range of Motion as the outcome measure. Considering a power of 80%, a two-sided significance level of 0.05 and a dropout rate of 10%, to detect a 10° difference between the two groups, a sample size of 52 patients will be needed. A standard deviation of 12° was determined by previous clinical trials [25,26].

The statistical analysis over the results will be performed by a blinded expert. Within and between-group comparisons will be performed by Two-sample t-test or one-way and two-way ANOVA analysis.

Data will be expressed as mean ± standard deviation (normally distributed data) or median and interquartile range (non-normally distributed data). The threshold for statistical significance will be set to P = 0.05. Missing data will be treated with the Last Observation Carried Forward method.

All statistical tests will be performed with Matlab (The MathWorks, Inc., Natick, Massachusetts, United States) software package.

## Discussion

This study aims to assess the effectiveness of a home-based telerehabilitation program for the improvement of physical function and clinical outcome following TKA.

In addition, patient satisfaction with the telerehabilitation solution (ReHub) is evaluated to explore whether it increases the adherence to the treatment among other improvements. Another important point of the study will be the evaluation of the costs of the rehabilitation process with the ReHub program compared to conventional rehabilitation and as a result, the economic effects it can have, particularly for the public health system.

The randomized controlled design, blinding of the individual performing the outcome assessments, and use of valid tools for the assessment of physical performance and muscle strength (TUG score and dynamometer-based measurements of muscle strength under isometric conditions, respectively) are notable strengths of the study.

However, the short duration of the intervention (4 weeks) and the lack of a long-term follow-up that could help determine with more precision whether telerehabilitation could have lasting benefits could be a limitation to our study. Also, the fact that the adherence of control patients to their exercise program is self-reported in an exercise diary, could affect the evaluation of this parameter.

Notwithstanding these limitations, demonstrating the effectiveness of a home-based telerehabilitation program for physical function and/or muscle strength improvement following TKA could have relevant implications for the post-surgical rehabilitation process. In fact, telerehabilitation solutions could facilitate access and adherence to health interventions, reduce health care costs (associated with supervision, facility provision, and transport of patients), and also contribute to social distancing when it becomes necessary as an infection control action. In addition, the results of the study could support the systematic incorporation of solutions like ReHub in post-surgical rehabilitation protocols, that should be tailored to the individual and collective needs.

## Trial Status

The first study participants were recruited into the trial in December 2019. The study was planned to end by April 2020, but recruitment was heavily altered due to the COVID-19 pandemic situation in Spain. Patient recruitment and data collection are ongoing and will continue until the required number of study participants is achieved.

## Data Availability

Data will be made available upon request to the corresponding author due to privacy or other restrictions.
